# A cornucopia of cryptic species - a DNA barcode analysis of the gobiid fish genus *Trimma* (Percomorpha, Gobiiformes)

**DOI:** 10.3897/zookeys.381.6445

**Published:** 2014-02-18

**Authors:** Richard Winterbottom, Robert H. Hanner, Mary Burridge, Margaret Zur

**Affiliations:** 1 Department of Natural History, Royal Ontario Museum, 100 Queen’s Park, Toronto, Ontario, M5S 2C6, Canada; 2 Department of Ecology & Evolutionary Biology, University of Toronto, 25 Willcocks Street, Toronto, Ontario, M5S 3B2, Canada; 3 Centre for Biodiversity Genomics, University of Guelph, Guelph, Ontario, N1G 2W1, Canada

**Keywords:** *Trimma*, gobies, mtDNA, COI, cryptic species, Indo-West Pacific ocean

## Abstract

A genetic analysis of partial mitochondrial 5’ cytochrome *c* oxidase I gene (DNA barcode) sequences of 473 specimens assigned to 52 morphological species (including four known, but not formally named, species) of the gobiid genus *Trimma* revealed the presence of 94 genetic lineages. Each lineage was separated by > 2% sequence divergence. Thus there were an additional 42 haplogroups recognizable as provisional candidate species given that a value of > 2% difference is typical of different species of fishes. Such a high degree of apparently different cryptic species is, in our experience, virtually unprecedented among vertebrates. These results have precipitated further morphological research in a few cases, which has uncovered subtle differences independently corroborating the genetic results. However, such efforts are limited by the dearth of traditional systematists available to undertake the necessary time-consuming, and highly detailed, morphological research. In some cases, the genetic results we present are consistent with, and confirm, minor taxonomic distinctions based on morphology and/or colour pattern. In other instances, what had been recognized as a single species consists of several genetic lineages - up to eight in, for example, what we have identified based on morphology as *Trimma okinawae*. The increase from 52 to 94 potential species in our sampling raises the predicted total number of species in this genus from about 110 to nearly 200 (versus the 73 valid described species currently recognized).

## Introduction

Descriptions of new species of the small (average 2 cm SL), often colourful, Indo-Pacific pygmy gobies of the genus *Trimma* Jordan & Seale, have exploded during the last 30 years or so – from 17 species recognized as being valid prior to Winterbottom’s (1984) publication to the 73 valid species recognized today. Numerous additional species of these coral reef inhabitants have been recognized as morphologically distinct from those already described, and await publication. One of us (RW) estimates the current number of known, morphologically distinctive, species to be about 110 (approximately 37 undescribed).

Previous studies of the 5’ mitochondrial cytochrome c oxidase subunit I (COI; the animal “DNA Barcode”) gene in other goby genera have frequently uncovered or documented hidden diversity (e.g. Baldwin et al. 2009, Tornabene et al. 2010, Victor 2010). Tornabene et al. (2010) studied western Atlantic members of *Bathygobius* Bleeker. At the time of their study, there were three recognized species, but matters were complicated by numerous proposed subspecies, and many nominal species. Nonetheless, their study revealed the presence of eight discreet genetic lineages, of which at least two do not appear to have available names (one of two lineages of *Bathygobius soporator* (Valenciennes), 2.9% difference; and one of two lineages of *Bathygobius geminatus* Tornabene et al., 2.1% difference). The mean interspecific genetic differences between the lineages in the western Atlantic varied between 2.1–17.2% (the latter value between *Bathygobius antilliensis* Tornabene et al. and *Bathygobius lacertus* (Poey)). Similarly, a study by Baldwin et al. (2009) on the Western Atlantic and Caribbean species of *Coryphopterus* Gill revealed 12 genetic lineages for which 11 species names were available. One described species (*Coryphopterus punctipectophorus* Springer) was not available for their study, and the single Curacao specimen that forms a separate lineage from *Coryphopterus alloides* Böhlke & Robins, is under further study and has not yet been formally named. The mean differences between lineages recorded by Baldwin et al. (2009) were generally greater than those recorded by Tornabene et al. (2010) for *Bathygobius*, ranging from 7.1% (*Coryphopterus hyalinus* Böhlke & Robins vs. *Coryphopterus personatus* (Jordan & Thompson)) to 27.9% (*Coryphopterus glaucofraenum* Gill vs. *Coryphopterus kuna* Victor). A similar situation at a smaller scale has been documented by Victor (2010), who found that *Elacatinus multifasciatus* (Steindachner) contained three genetic lineages, two of which he described as new. Both of these differed by about 11% from specimens from the type locality of *Elacatinus multifasciatus*, but only *Elacatinus rubrigenis* Victor exhibited a trenchant morphological difference. The other new species, *Elacatinus panamensis* Victor, is very similar in external appearance to *Elacatinus multifasciatus*, yet is separated from *Elacatinus rubrigenis* by only 3% of the COI gene, suggesting a lack of congruence between genetic and morphological divergence in this group. In earlier studies of *Elacatinus*, Taylor and Hellberg (2005, 2006) suggested that, for this genus of 34 currently recognized species (Froese and Pauly 2013), speciation was consistent with a model of regional but recurrent adaptive radiations driven by a combination of colouration and ecological differentiation. However, we note that Hsu et al. (2011) provide evidence that, at least in *Siganus* Forsskål, 1775, species defined by colour differences do not necessarily equate with genetic lineages.

Such cryptic diversity among small reef fishes is not, apparently, confined to gobies. Baldwin et al. (2011) described seven new species of the labrisomid *Starksia* Jordan & Evermann following a barcode analysis of 13 of the currently recognized Caribbean species. Their Neighbour-Joining network (Baldwin et al. 2011, fig. 1) shows numerous other apparently distinct genetic lineages that may well represent discreet species among their samples, although most of these had only a few available specimens. In a more wide-ranging biogeographical study of fishes, Hubert et al. (2012) compared 141 apparent con-specifics between the Indian and Pacific oceans. They found no difference in COI in 44% of the species, but uncovered two (47% with genetic differences ranging from 1−12%) or more (9% with distances varying between 3–22%) haplogroups in the other con-specifics. In a study of 207 marine fishes from Australian waters, Ward et al. (2005) reported an average within species variation of 0.4% and a between species variation of 9.9%.

In order to document the extent of cryptic diversity in *Trimma*, we performed a barcode analysis based on 473 specimens of this genus, most of them from the fish tissue collections of the Royal Ontario Museum.

## Materials and methods

The ROM specimens used in our analysis (catalogue number prefixed by “T”) were collected using overdoses of various anaesthetics (e.g. clove oil, quinaldine), and the whole fish was placed in a vial containing either a saturated salt solution (Seutin et al. 1990; specimens collected prior to 1998) or 95% ethyl alcohol (after 1998). These specimens were subsampled for genetic analysis. In collections made prior to 2006, the samples retained for genetic analysis were seldom photographed immediately after collection, although photographs of a specimen from the same lot as the tissue specimen were frequently taken. In the collections made in 2011, all tissue specimens were photographed. Values given in the tables are rounded to the nearest decimal point. We use the term “variance” to denote the percentage of sequence divergence within a group. For undescribed but known species, we use a system of numbering the species prefixed by ‘RW’. These numbers are unique and consistent for a given undescribed species pending its formal description. The system allows the specimens to be catalogued in museum collections, and obviates the problems frequently found in checklists and books on Indo-Pacific fishes where species are simply numbered sequentially, with the same number nearly always denoting different species between different, and sometimes the same, author(s).

Voucher specimen information and digital images (where applicable) were deposited in the Barcode Of Life Database (BOLD, http://www.barcodedsystems.org; Ratnasingham and Hebert 2007) following recommendations of the FISH-BOL campaign (Ward et al. 2009) and related collaborators protocol (Steinke and Hanner 2011) in a publicly accessible data project (titled “Trimma12”; DOI: 10.5883/DATASET-TRIMMA12).

DNA was extracted using a Qiagen DNeasy Blood & Tissue Kit (QIAGEN) following the manufacturer’s instructions with some exceptions: after adding AW2, spin columns were dried through a final centrifugation at 17,000× g for 5 minutes; sample DNA was eluted with 50 µL of AE buffer and centrifuged at 6,000× g for 1 minute, and the same 50 µL of AE buffer was then re-eluted with a final centrifugation at 6,000× g for 1 minute in order to increase the DNA concentration. Each 12.5 µL PCR reaction consisted of 2 µL of template DNA, 6.25 µL 10% trehalose, 2 µL ddH2O, 0.625 µL MgCl2 [50 mM], 0.0625 µL dNTPs [10 mM], 0.06 µL Platinum Taq (Invitrogen), 0.10 µL [0.01 mM] each of the universal fish COI cocktail primers C_FishF1t1 and C_FishR1t1 (Ivanova et al. 2007) and 1.25 µL 10X PCR buffer (Invitrogen). PCR thermocycling conditions were an initial hot start of 94 °C for 2 min, 25 cycles of denaturation at 94 °C for 30 s, annealing at 52 °C for 40 s and extension at 72 °C for 1 min, with a final extension at 72 °C for 10 min. PCR products were visualised using 2% agarose gel E-Gel96 Pre-cast Agarose Electrophoresis System (Invitrogen). Only amplicons with single, intense bands were sequenced.

Each sequencing reaction consisted of 1 µL of PCR product along with 1 µL BIG DYE 3.1 reagent (Applied Biosystems, Inc), 1 µL M13F/M13R primer (Messing 1983), 10 µL ddH20 and 1 µL 5X sequencing buffer (Invitrogen). The thermocycling profile was an initial hot start 96 °C for 2 min, followed by 30 cycles of denaturation at 96 °C for 30 s, annealing at 55 °C for 15 s, and an extension at 60 °C for 4 min. PCR products were bi-directionally sequenced and run on an ABI 3730 capillary sequencer (Applied Biosystems). Sequencher 4.05 (GeneCodes) was used to trim primers, assemble and manually edit bidirectional contigs from raw trace files.

Sequence assemblies and supporting electropherogram “trace” files were uploaded to BOLD (and submitted to GenBank, accession numbers: KJ202257–KJ202608) and combined with other available *Trimma* sequences using a Hidden Markov Model alignment of translated COI amino acid sequences (Ratnasingham and Hebert 2007). Aligned sequences of > 500 bp in length were used to generate pairwise or p-distances to infer a Neighbour-Joining phenogram of sequence divergences using BOLD in order to provide a visual depiction of the barcode variation among and between species. Sequence data from an updated data set were also parsed into molecular operational taxonomic units (MOTUs) using the RESL (Refined Single Linkage Analysis) algorithm and subsequently annotated with Barcode Index Numbers (BINs), as implemented on version 3 of BOLD (Ratnasingham and Hebert 2007). This approach combines single linkage clustering and Markov clustering to recognize gaps in sequence space that correlate with species boundaries by optimizing MOTU partitions using the Silhouette index and uniquely labelling each MOTU with a Barcode Index Number or BIN (detailed in Ratnasingham and Hebert (2013)). The value of 2% used here is approximately equivalent to the more sophisticated RESL analysis used to distinguish haplogroup clusters that are subsequently enumerated with Barcode Index Numbers or BINs as promulgated by Ratnasingham and Hebert (2013, see above).

## Results

The presentation of the results follows the sequence of appearance of the species names in the main barcode phenogram (Fig. 1), except where there are several haplogroups differing by > 2% sequence divergence under a single species name, or where a few such names alternate within such groups. In these cases, all such haplogroups are discussed under a single heading. Species which appear to form well defined monospecific entities and which have < 2% within species variance are listed below, and are not discussed further. These include (in the order in which they appear in Fig. 1): *Trimma maiandros* Hoese et al., *Trimma rubromaculatum* Allen & Munday, *Trimma habrum* Winterbottom, *Trimma anaima* Winterbottom, *Trimma imai* Suzuki & Senou, *Trimma agrena* Winterbottom & Chen, *Trimma hotsarihiensis* Winterbottom, *Trimma sheppardi* Winterbottom, *Trimma haimassum* Winterbottom, *Trimma* RW sp. 24, *Trimma yanoi* Suzuki & Senou, *Trimma papayum* Winterbottom, *Trimma kudoi* Suzuki & Senou, *Trimma halonevum* Winterbottom, *Trimma tauroculum* Winterbottom & Zur, *Trimma haima* Winterbottom, *Trimma* RW sp. 97, *Trimma* RW sp. 98, *Trimma preclarum* Winterbottom, *Trimma necopinum* (Whitley), *Trimma mendelssohni* (Goren), *Trimma corallinum* (Smith), *Trimma cana* Winterbottom, *Trimma fucatum* Winterbottom & Southcott, *Trimma annosum* Winterbottom, *Trimma hoesei* Winterbottom, *Trimma randalli* Winterbottom & Zur, and *Trimma flavatrum* Hagiwara & Winterbottom. However, it seems significant that, of these 27 taxa, all but six are represented from a single geographic locality in our material. The exceptions are: *Trimma anaima* (2, Timor and Raja Ampat), *Trimma halonevum* (8, Bali, Raja Ampat, Timor and Vanu Atu), *Trimma annosum* (14, Great Barrier Reef, Palau, Raja Ampat and Taiwan), *Trimma haimassum* (6, Rabaul and Raja Ampat), *Trimma hoesei* (4, Palau and Raja Ampat), and *Trimma yanoi* (6, Palau, Rabaul and Raja Ampat). All the others consist of more problematic species and groups discussed below, and these, too, are treated in the order that they appear in colour-coded labeled blocks in Fig. 1. The results are presented under subheadings where we felt the complexity of the analysis warranted such treatment for the sake of clarity.

**Figure 1.**
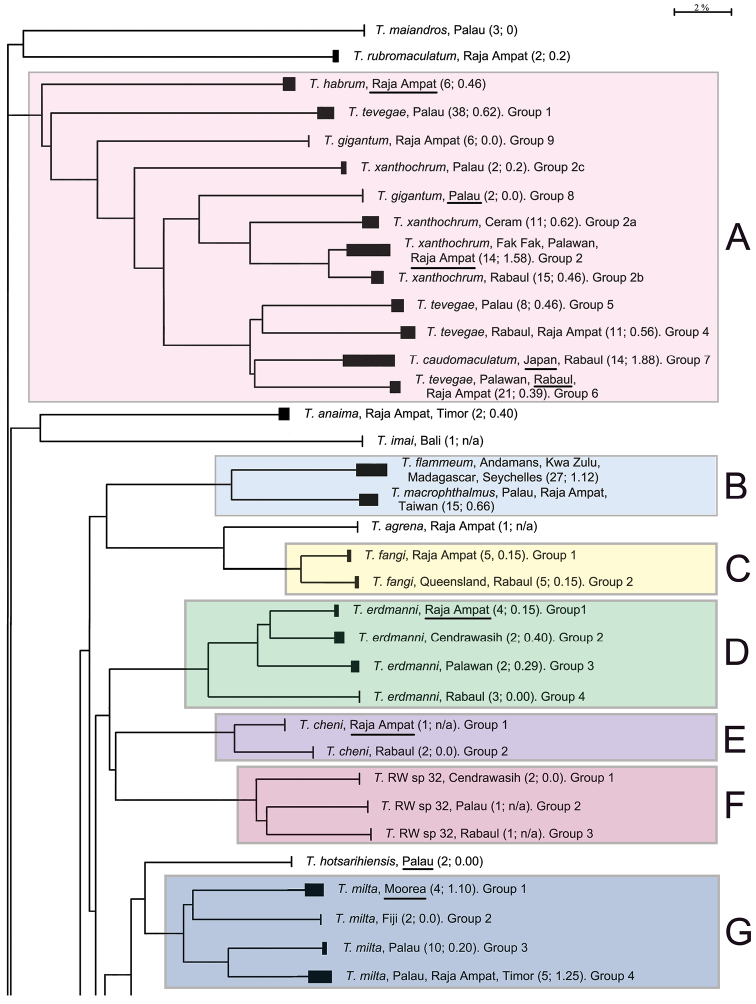
Condensed Neighbour-Joining network of the COI gene based on an analysis of 473 specimens of *Trimma*. Solid bars represent approximate within group variation. Species names/numbers are followed by the locality of the specimens, with the number of specimens followed by the percentage variation within the group in parentheses. Scale bar: 2% genetic distance. Coloured boxes and their alphabetical notations refer to the sequential groups discussed in the text. Type localities for each species are underlined where such samples were available. "Queensland" is used in lieu of "Great Barrier Reef".

**Figure 1.**
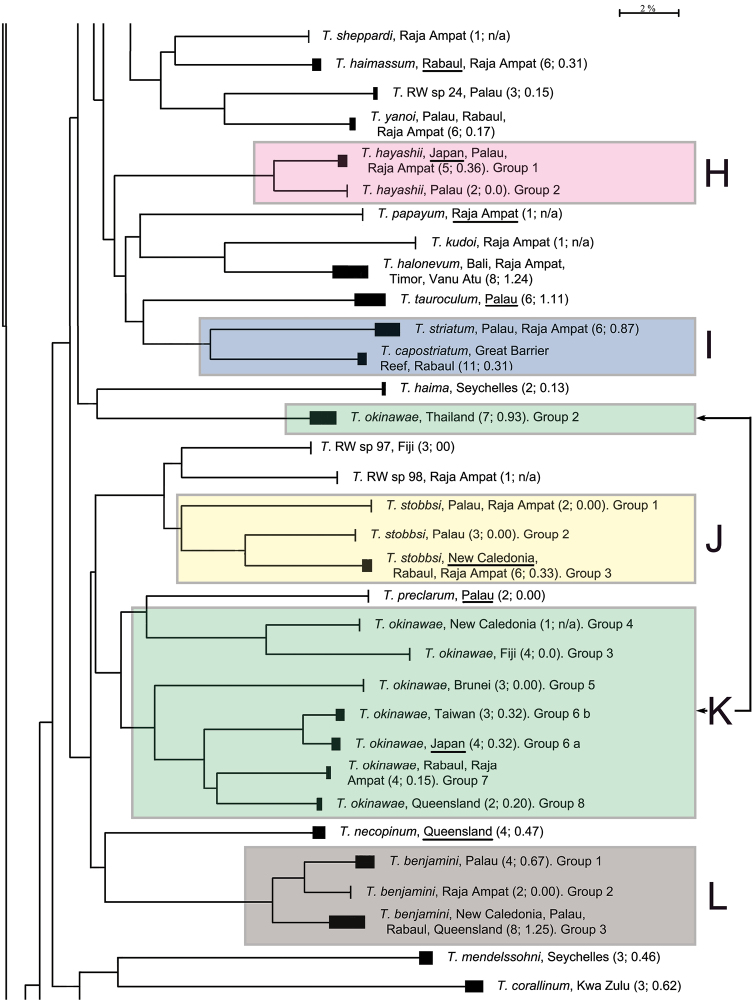
Continued.

**Figure 1.**
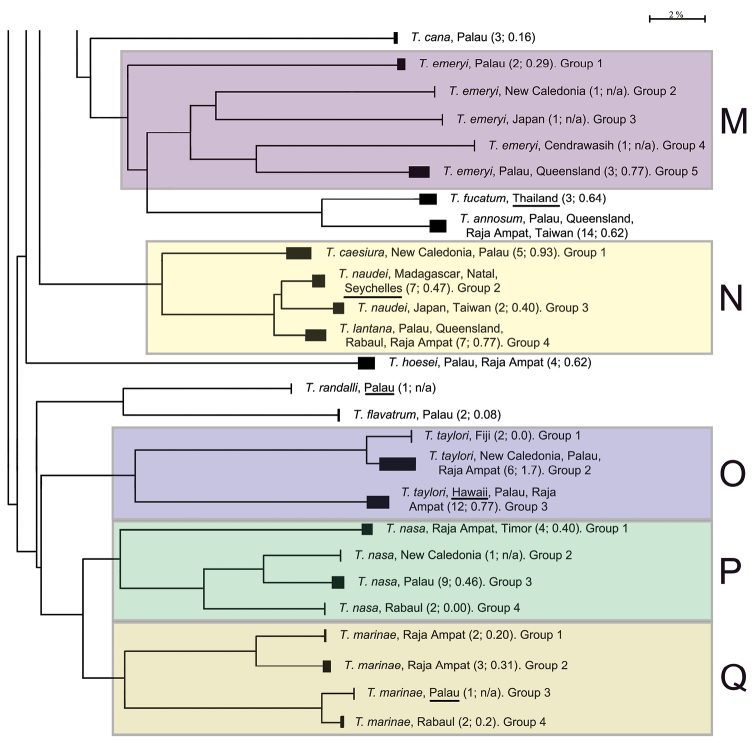
Concluded.

### The *Trimma tevegae* group

**Figure 2.**
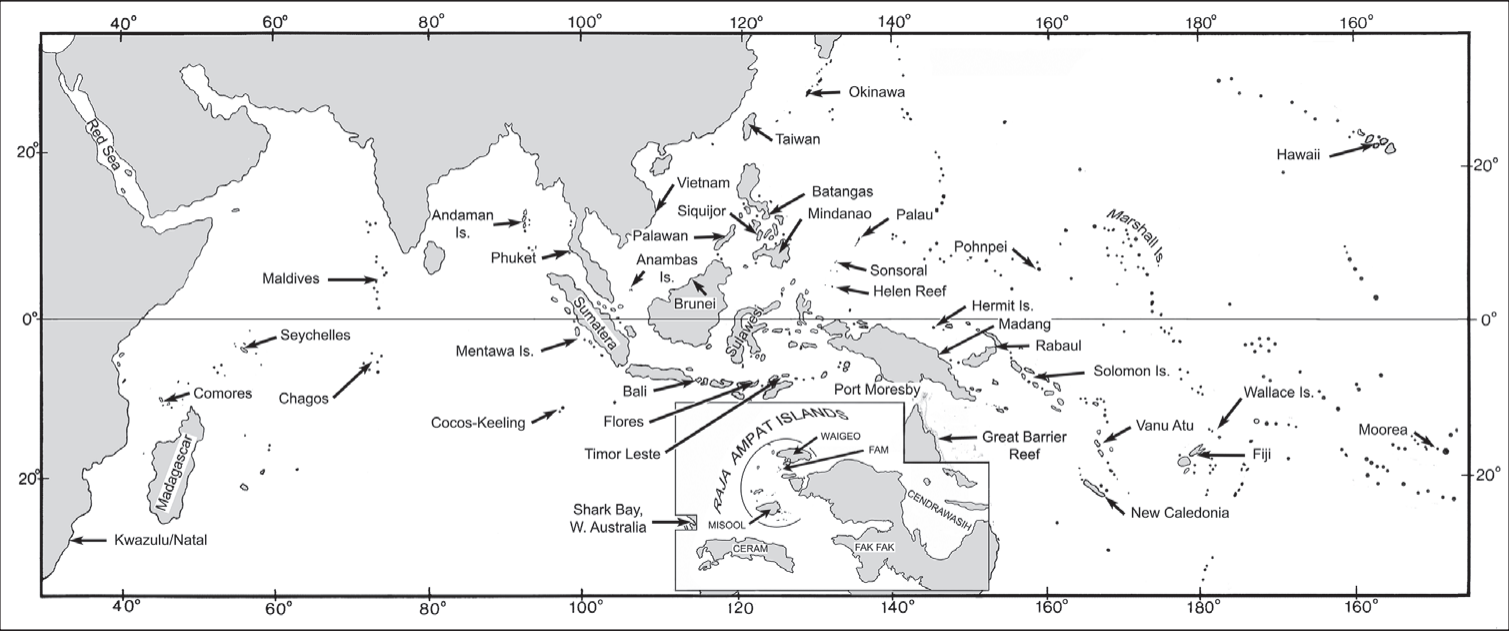
Map of the Indo-Pacific, showing localities where samples were collected. Inset - details of the Raja Ampat islands and Bird’s Head region of Papua New Guinea.

**Figure 3.**
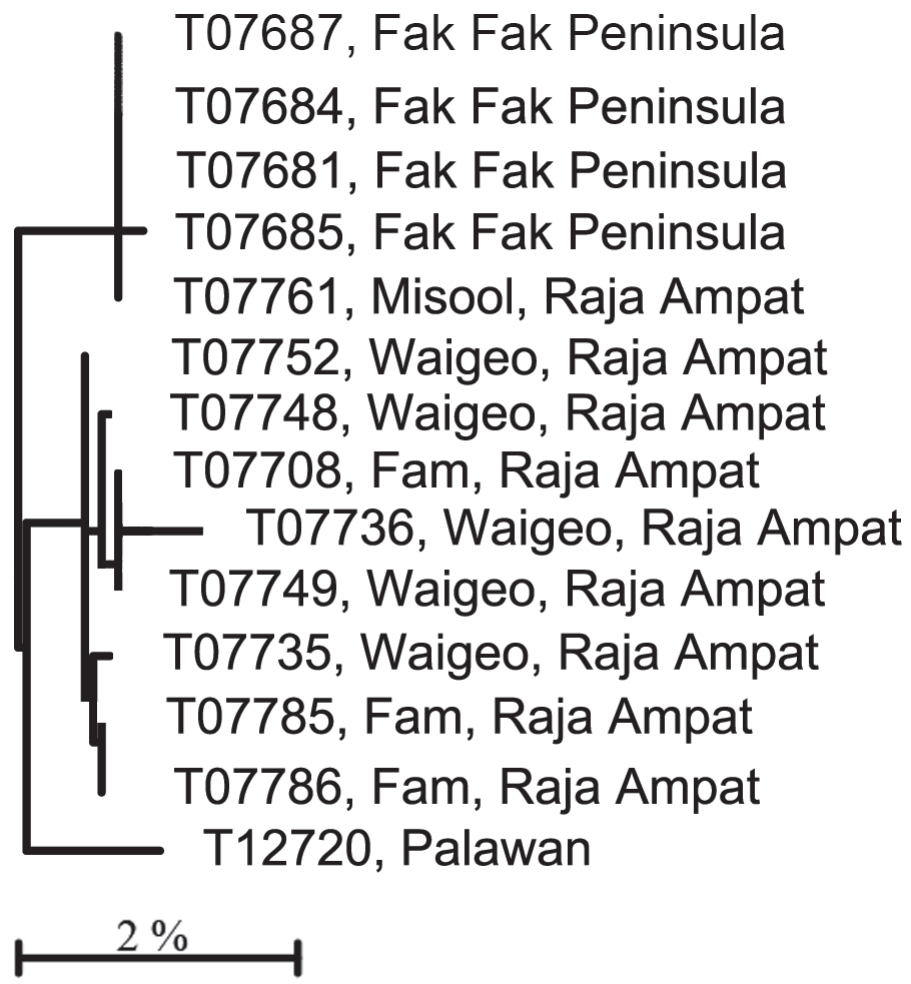
Relationships of 14 specimens of the *Trimma xanthochrum* Group 2, based on COI.

**Figure 4.**
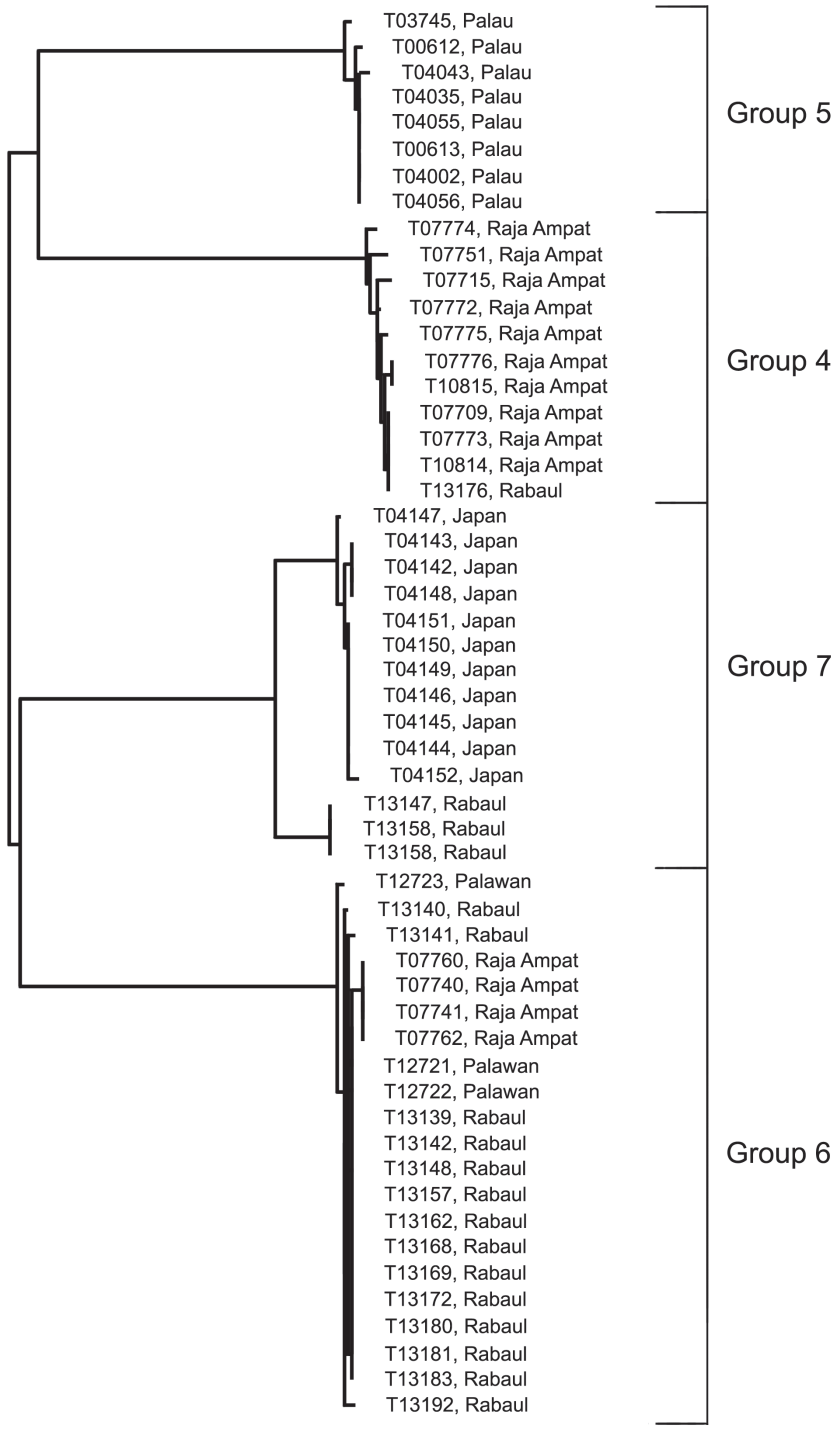
Relationships of 54 specimens of the *Trimma tevegae* – *Trimma caudomaculatum* group based on COI.

Figs 1A, 2–4, 13; Tables 1–2

**Note.** This is the most taxonomically complex of all the groups within *Trimma*. It contains four nominal species, all of which appear to be valid (Winterbottom 2011), and is made up of at least 11 different, and often widely divergent, haplogroups in our barcode analysis (Fig. 1). The species for which the group is named, *Trimma tevegae* Cohen & Davis, 1969, was described from specimens collected at the Dawapia Rocks in Rabaul Harbour, New Britain (see map, Fig. 2). Yoshino and Araga (1975) described *Trimma caudomaculatum* (as *Trimma caudomaculata*) from the Okinawa Island, part of the Ryukyu Islands, Japan. Winterbottom (2005a) compared these two nominal taxa morphologically based on material from throughout the western Pacific (including type specimens of both nominal species). He concluded that, while there was considerable morphological and colour variation present in the material, all the specimens represented a single species, since he could find no consistent patterns in the variation he described. Winterbottom and Zur (2007) described *Trimma gigantum* from relatively deep collections (57–73 m) made in Palau by Patrick Colin. They compared their new species to *Trimma fishelsoni* (Goren), currently known only from the Red Sea, and did not include any detailed reference to *Trimma tevegae*, since their new species clearly differed from other members of that complex in lacking any trace of a dark caudal blotch. In 2011, Winterbottom described *Trimma xanthochrum* from the Raja Ampat Province of Indonesia. In the discussion section of that description, he reversed his opinion (Winterbottom 2005a) that *Trimma tevegae* and *Trimma caudomaculatum* represented the same species, and hinted at morphological and colour differences that could be used to separate them from each other and from *Trimma xanthochrum*. He also suggested, based on barcode analyses done up to that time, that there were several other haplogroups in the *Trimma tevegae* complex that warranted more detailed morphological investigation to see whether any characters could be found to support this limited genetic evidence. One problem that he mentioned was the lack of genetic material from the type locality of *Trimma tevegae*, a situation since rectified by a two week collecting trip he made to Rabaul, New Britain in November, 2011.

**Analysis.** A) Although the most basal taxon associated with this group is *Trimma habrum* (Fig. 13A), we have not included it in the following analysis. The most phenetically divergent member of *Trimma tevegae* group in terms of its COI gene is from Palau, and was originally identified as *Trimma tevegae*. It is very similar in overall morphology to the rest of the *Trimma tevegae* group which possess a dark caudal blotch (Fig. 13B), and is listed as Group 1 in Fig. 1, section A. It differs from all the other haplogroups by a minimum of 17.7% of the COI base pairs (see Table 1).

**Table 1.** Results from a barcode analysis of 142 specimens of *Trimma tevegae* group, giving number of specimens per group, the maximum variation within each group, followed by the minimum distances between groups (as percentages). Specific names under Group # are the names used in the networks; locality abbreviations are: FF, Fak Fak Peninsula; P, Palawan; Ra, Rabaul; and RA, Raja Ampat.

Group #	Locality	n	Variation	Minimum distance between groups									
				2	2a	2b	2c	4	5	6	7	8	9
1 (as *Trimma tevegae*)	Palau	38	0.6	17.7	21.3	19.3	19.7	21.5	21.1	22.4	20.8	21.6	18.1
2 (as *Trimma xanthochrum*)	FF/P/RA	14	1.6	–	7.7	2.5	16.1	14.7	14.0	14.2	13.8	10.7	15.2
2a (as *Trimma xanthochrum*)	Ceram	11	0.6		–	7.8	16.3	15.8	14.8	16.2	13.9	12.0	17.3
2b (as *Trimma xanthochrum*)	Rabaul	15	0.5			–	16.7	16.2	14.6	15.1	14.4	10.3	14.6
2c (as *Trimma xanthochrum*)	Palau	2	0.0				–	15.8	14.9	15.2	14.6	15.6	15.9
4 (as *Trimma tevegae*)	RA/Ra	11	0. 6					–	9.2	9.5	9.1	15.1	17.0
5 (as *Trimma tevegae*)	Palau	8	0.5						–	9.5	9.1	14.7	18.5
6 (as *Trimma tevegae*)	P/Ra/RA	21	0.4							–	7.9	14.5	16.8
7 (as *Trimma tevegae*)	Japan/Ra	14	1.7								–	14.1	16.8
8 (as *Trimma gigantum*)	Palau	2	0.0									–	16.7
9 (as *Trimma gigantum*)	RA	6	0.0										–

**Comments.** Further morphological work initiated as a result of this study suggests that Group 1 is separable from the others based on a combination of four blotches (made up of melanophores) around the eye at 3, 4:30, 6 and 9 o’clock positions (only visible in well-preserved material, although the elongate blotch below the eye is usually apparent), the second spine of the first dorsal fin somewhat elongate, reaching to the base of the 4^th^ to 7^th^ ray of the second dorsal fin when adpressed, 13 unbranched pectoral-fin rays, and a single row of 6–7 cheek scales. The freshly collected colour pattern is based on a colour slide of a single, damaged 14.4 mm SL female specimen (Fig. 13B) that is probably of this haplogroup (i.e. it fits the above morphological criteria above). It is a relatively drab fish, being darkish red below the midlateral septum and lighter above. In preservative, the dorsum is darkened by melanophores, especially around the scale pockets, and by a scattering of large brown chromatophores which decrease in number towards the midline. The ventrum has a slightly greater concentration of large brown chromatophores, and few, if any, melanophores, and the combination gives the impression of a moderately dark fish with a somewhat lighter diffuse lateral stripe. It appears to be a small species (maximum recorded SL of 17.3 mm), and is only present in our analysis from the main islands of Palau. All but one of the 38 specimens we processed are from a single collection. Further work is underway to formally describe this new species (Winterbottom in prep.).

B) Morphologically, specimens identified as *Trimma gigantum* can easily be distinguished from the other members of the group by the lack of a large dark blotch at the base of the caudal peduncle. However, the barcode analysis widely separates two distinct geographic entities, both originally identified as this species. The two specimens from Palau used in our analysis were collected together with the holotype and 21 paratypes at Uchelbeluu Reef in Palau (Winterbottom and Zur 2007, labeled as *Trimma gigantum* Group 8 in Figs 1, 13E). These specimens nest phenetically with the members of the *Trimma xanthochrum* subgroup (q.v.), but are separated from them by a minimum distance of 10.3% of the COI base pairs. The second lot of samples provisionally identified as this species (Fig. 13C; 6 specimens from the Raja Ampat islands of Indonesia, labeled as *Trimma gigantum* Group 9 in Fig. 1A) show no variation in COI among themselves, but are separated from all other haplogroups in the group by a minimum of 14.6% sequence divergence.

**Comments.** A preliminary analysis of morphology between these two haplogroups did not reveal any obvious differences, and further work is clearly necessary if we are going to be able to distinguish between them (especially preserved museum material).

C) Four haplogroups are identifiable among what we call the *Trimma xanthochrum* subgroup (Fig. 1A). Of these four, *Trimma xanthochrum* Group 2c, from Palau (Fig. 13D), is the most different in its COI (a minimum of 16.1% sequence divergence from any other group) and in having a brownish, rather than yellow, overall colouration. *Trimma gigantum* from Palau (see above) is placed phenetically at the next node, forming the congruent level group with groups 2a (Ceram – no image available), 2 (Fak Fak Peninsula, Raja Ampat – the type locality of *Trimma xanthochrum*, and Palawan; Fig. 13F) and 2b (Rabaul; Fig. 13G). Group 2a (Ceram) is separated by a minimum of 7.8% sequence divergence from its nearest neighbour, and Group 2b shows a 2.5% minimum difference from specimens included here as Group 2. The relationships of this latter group are complex, and, based on current sampling, show some geographic variation (Fig. 3, Table 2). Specimens from southern Misool Island and from the southern side of the Fak Fak Peninsula vary from each other by 0.2%, and by a minimum of 1.1% from others in this assemblage. The eight specimens from the Raja Ampat Islands to the north have a variance of just 0.4%, and are separated from a single specimen from Palawan by a minimum of 1.2%. Group 2 differs from Group 2b (15 specimens from Rabaul, variance 0.5%) by a minimum value of 2.5%.

**Table 2.** Results from a barcode analysis of 14 specimens of *Trimma xanthochrum* Group 2, giving maximum variation within each group followed by the minimum and maximum distances between groups (as percentages). These values are typical of intraspecific barcode divergences among fishes.

Locality	n	Variation	Min./max. distance between groups	
			Raja Ampat	Palawan
Fak Fak/Misool	5	0.2	0.9/1.1	1.6
Raja Ampat	8	0.5	0	1.2/1.6
Palawan	1	n/a	-	0

**Comments.** Morphologically, all these fishes possess a large dark blotch on the caudal peduncle and base of the caudal fin, a generally yellowish to brownish body, and have two or more sensory papillae in vertical rows beneath the eye and transversely in the interorbital row (part of row *p*: see description and figures of *Trimma xanthochrum* in Winterbottom 2011). No further morphological work has yet been undertaken to search for characters that might support the separation of the *Trimma xanthochrum* haplogroups.

D) The *Trimma tevegae* – *Trimma caudomaculatum* subgroup contains four haplogroups (identified as *Trimma tevegae* Groups 4, 5, 6 and 7 in Figs 1 and 4). Group 4 (Rabaul and Raja Ampat, 11 specimens, variance 0.6%; Fig. 13I) differs by a minimum of 9.2% from Group 5 (Palau, 8 specimens, variance 0.5%; Fig. 13H); these two together differ from the remaining two haplogroups by a minimum of 9.1% (see Table 1). The remaining two haplogroups, Group 6 (Palawan, Rabaul and Raja Ampat, 21 specimens, 0.4% variance; Fig. 13K) differs from Group 7 (Japan and Rabaul, 14 specimens, 1.9% variance; Fig. 13J) by a minimum of 7.9%. There is virtually no geographic structure discernible among the samples in Group 6, which is somewhat surprising to us given the relatively extensive geographic distribution of the samples.

**Comments.** There is currently no reliable way to distinguish between Groups 4 and 5 based on morphology despite fairly extensive data analyses, and further work is needed. The fishes in Group 6 differ morphologically from most others in the complex in having a relatively short second dorsal spine (reaching posteriorly between the interspace between the two fins to the base of the second dorsal-fin ray when adpressed), a single papilla in rows 3 and 4 below the eye (row *c*), row *p* in the interorbital region also consisting of single papillae, nearly always 13 unbranched pectoral-fin rays, usually a diffuse dark blotch just behind the symphysis of the lower jaw, and larger, darker brown spots among the brown chromatophores on the dorsal surface of the snout. In all these characters, these specimens match the types of *Trimma tevegae*, and we are relatively confident that the Group 6 haplotype represents the true *Trimma tevegae*. In the case of *Trimma caudomaculatum* (Group 7) there appears to be a clear cut difference of between 1.7–1.9% between the Japanese samples (11 specimens, variance 1.7%) and the three specimens from Rabaul (0.0% variance). We have here simply regarded these populations as the same species, since the maximum difference value between them is < 2%. However, we note that the RESL algorithm places the Japanese and Rabaul populations in separate BINs – the only difference between the two criteria (i.e. < 2% threshold vs RESL) that was present in our data set. Our specimens from Japan were collected off the north coast of Ie-jima Island, which lies just off the northern margin of Okinawa Island, the type locality of *Trimma caudomaculatum*. They appear to agree with the description and type specimens of *Trimma caudomaculatum*, a species apparently characterized in life by a bright blue lateral stripe from the upper orbit to the upper peduncle, another in the mid-dorsal region from the anterior snout to the base of the dorsal fins, a blue bar on the cheek just below the eye, and a mauve to magenta flush above and below the caudal spot which continues posteriorly onto the basal half of the caudal fin. This species has a very elongate second spine in the first dorsal fin, which often reaches to or beyond the end of the second dorsal fin when adpressed, and the head papillae are as in *Trimma tevegae* (Group 6). In preserved specimens, the marking under the eye is normally present as a well-developed dark stripe, the top of the snout is very dark and heavily pigmented, and there is often scattered dark pigment on the undersurface of the head. Specimens recently collected by Mark Erdmann in Timor Leste and at Port Moresby, Papua New Guinea, include two specimens each for DNA analysis (both samples received too late for inclusion here) of what appear, both in morphology and in live colour pattern, to be *Trimma caudomaculatum*. Live specimens exhibiting the blue lateral stripe have been photographed at several localities in the western Pacific, and may be this species, or there may prove to be more than one taxon involved. Large specimens of both Group 4 and Group 5 may have blue lateral and dorsal stripes. In addition, there is undoubtedly more than just a single species of this group in Japanese waters (see, e.g. the images in Senou et al. 2004, and various websites such as http://fishpix.kahaku.go.jp/fishimage-e/search.html). Among the specimens from Raja Ampat, tissue samples falling into Group 2 were twice collected with tissue voucher specimens from Group 4, and once together with the tissue voucher from Group 6, so these forms are syntopic. All Palau specimens were from contiguous island groups except for Group 5, which consists of three specimens from the main islands of Palau (from both the east and the west coasts), and five specimens from Helen Reef in the South West Islands, some 570 kilometers (350 miles) to the south-west. Group 5 was the only haplogroup represented in the samples from Helen Reef, whereas the main Palauan islands had representatives of Groups 1, 2c and 5. Variance for Group 5 was 0.5%, without any correlation with geographic origin. Among the Palauan samples, the only coincident sampling involved the simultaneous collection of a specimen from each of Groups 1 and 2c at 20–28 m on Uchelbeluu Reef near Koror on the east coast of the main islands. The collections made at Rabaul differed from those made elsewhere in that each individual site sampled was kept separate from all the others made during that dive, with the precise depth recorded. Each such collection was made at a specific substation (overhang, small cave, fissure, etc.) on the reef. We here report only on the specimens from which tissues were taken because there are still some difficulties in separating preserved specimens into groups based on morphology. Four haplogroups were present at Rabaul, although only three were collected at Dawapia Rocks (the type locality of *Trimma tevegae*). The fourth haplogroup represented, here reported as *Trimma tevegae* Group 4 (otherwise known at present only from Raja Ampat), was represented by a single specimen from Little Pigeon Island, some 18 km ESE of Dawapia Rocks and outside Simpson Bay. The three haplogroups at the type locality were *Trimma xanthochrum* Group 2, *Trimma caudomaculatum* Group 7 and *Trimma tevegae* Group 6. All three tissue samples of *Trimma caudomaculatum* were taken on the same dive, but at three different places and depths on the reef. Tissue samples of *Trimma tevegae* Group 6 were collected at two of these same substations, so these two haplogroups are syntopic at the micro-scale. Although tissue samples of *Trimma tevegae* Group 6 (n=14) and *Trimma xanthochrum* Group 2 (n=15) were not retained from exactly the same microhabitats, morphological identifications of the non-tissue specimens suggests that the two do co-occur in the same micro-habitat on occasion. Depth information based solely on the tissue samples indicates that *Trimma tevegae* Group 6 generally occurs at 13–29 m (mean 21 m), and that most *Trimma xanthochrum* Group 2 are found a little deeper (20–45, mean 32 m).

### The *Trimma flammeum*/*Trimma macrophthalmus* group

Fig. 1B

**Analysis.** *Trimma flammeum* (Smith) (variance 1.1%) and *Trimma macrophthalmus* (Tomiyama) (variance 0.7%) differ from each other by a minimum of 9.0% sequence divergence. Morphologically, and in colour pattern, these two species are virtually identical with only a slight variation in colour pattern (Winterbottom and Hoese in prep.), and their status as separate species has been unsupported empirically.

**Comments.** These two species are well represented in terms both of number of specimens (27 and 15 respectively) and in breadth of geographic range. The minimum 9.0% difference between the two haplogroups, suggests that the two are, in fact, distinct species. The only morphological difference that has been noted is that the spots on the pectoral fin base of *Trimma flammeum* are red in life and pale in preservative, while those in *Trimma macrophthalmus* are reddish-brown or dusky in life and dark in preservative (Winterbottom and Hoese in prep.). *Trimma flammeum* ranges from the south-western Indian Ocean (Kwazulu/Natal) to the Andaman Islands in the north-eastern Indian Ocean, while *Trimma macrophthalmus* occurs from the Cocos-Keeling Islands in the south-eastern Indian Ocean to Fiji in the east and north to Japan. It is currently unknown which species, if either, occurs at the Mentawa Islands off the west coast of Sumatera, as well as on Sumatera itself. These areas lie in the potential contact zone between the two species.

### The *Trimma fangi* group

Fig. 1C

**Analysis.** The 10 specimens in this group are all currently identified as *Trimma fangi* Winterbottom & Chen on the basis of morphology and colour pattern. Our barcode analysis, however, distinguishes two haplogroups separated by 3.5% sequence divergence, one from Raja Ampat (variance 0.2%) and the other from the Great Barrier Reef and Rabaul (variance 0.2%).

**Comments.** The type locality for this species is the Anambas Islands, in the South China Sea between mainland Malaysia and the island of Borneo, and we have no specimens with genetic material available for genetic analysis from this area. It is therefore unknown whether the specific name applies to either of the haplogroups, or whether it applies to a third group. Further morphological work is needed to clarify the situation.

### The *Trimma erdmanni* group

Figs 1D, 5; Table 3

This species was recently described based on specimens collected in the Raja Ampat islands by Winterbottom (2011), who commented that specimens attributable to this species were known from Sulawesi, the Hermit Islands, and Madang, and that there were photographs of specimens from Palawan, Mindanao and Batangas in the Philippines. Since that time, we have accumulated specimens identified morphologically as *Trimma erdmanni* from Cendrawasih Bay, Palawan and Rabaul in addition to the tissue specimens from the type locality in Raja Ampat.

**Analysis.** The specimens from Raja Ampat (Group 1; 4; 0.2% variance) are phenetically closest to those from Cendrawasih (Group 2; 2; 0.4% variance) but differ by 4.5% (Table 3). The next closest are the specimens from Palawan (Group 3; 2; 0.3% variance) which differ from these two groups by a minimum of 5.1%, followed by those from Rabaul (Group 4; 3; 0.0% variance), with a minimum distance of 8.4%.

**Figure 5.**
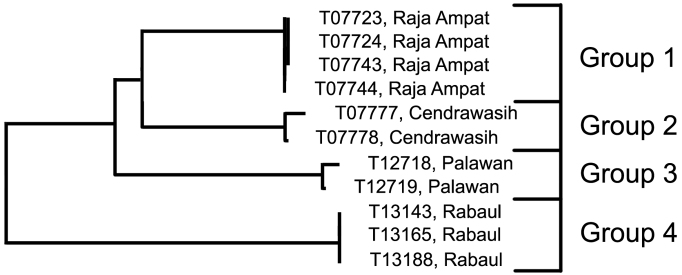
Relationships of 11 specimens of the *Trimma erdmanni* group based on COI.

**Table 3.** Results from a barcode analysis of 11 specimens of *Trimma erdmanni*, giving maximum variation within each group followed by the minimum distances between the groups (as percentages).

Group#	Locality	n	Variation	Min. Distance		
				2	3	4
**1**	Raja Ampat	4	0.2	4.5	5.1	8.4
**2**	Cendrawasih	2	0.4	0	6.5	10.1
**3**	Palawan	2	0.3	–	0	10.6
**4**	Rabaul	3	0.0	–	–	0

**Comments.** Mark Erdmann (pers. comm.) has informed us of several subtle colour and ecological differences he observed among specimens initially identified as this species, but these have yet to be quantified and compared to the barcode results. Additional morphological data needs to be gathered and analyzed along with the colour differences.

### The *Trimma cheni* group

Fig. 1E

This species was also recently described by Winterbottom (2011) from Raja Ampat, with additional records from the Philippines, Palau, Flores, Sulawesi and Ceram. Tissue specimens were only available from Raja Ampat (n=1) and from Rabaul (n=2), where the barcode analysis found two haplogroups differing by 4.5% sequence divergence. The specimen from Raja Ampat (Group 1) was collected with four paratypes of the species, and we assume that this specimen belongs to the same haplogroup as the holotype.

### The *Trimma* RW sp. 32 group

Fig. 1F

This informal name has been used for an undescribed species, characterised by a very large ocellated black spot in the first dorsal fin. Specimens with this distinctive marking are known from various localities in the western Pacific, including the Philippines and Wallace Island, as well as the tissue samples analysed here from Cendrawasih (Group 1; 2; 0.0% variance), Palau (Group 2; 1; n/a) and Rabaul (Group 3; 1; n/a). According to our analysis, each of these localities contains a distinct haplogroup, with samples from Cendrawasih differing from the others by a minimum of 7.4% sequence divergence, while the Palau and Rabaul specimens differ by a minimum of 7.1%. This clearly suggests that any formal descriptions of new species from within this group should be based on specimens from a single geographic locality, and that comparative material from other localities be examined minutely for potential morphological variations.

### The *Trimma milta* group

Figs 1G, 6; Table 4

**Figure 6.**
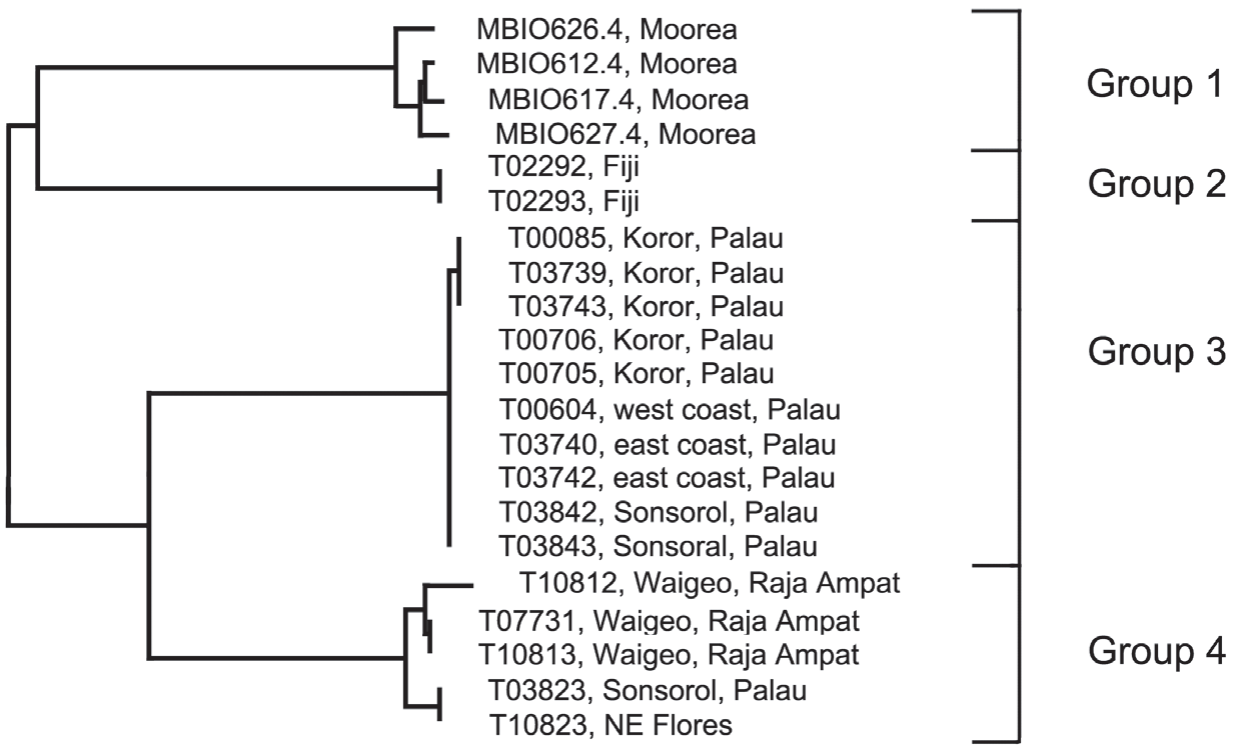
Relationships of 21 specimens of the *Trimma milta* group based on COI.

**Analysis.** The *Trimma milta* Winterbottom group contains four discreet haplogroups (Figs 1, 6). Group 1 (intra-group variation 1.1%) is from the type locality (Moorea, Society Islands), and differs by 7.9% from Group 3 (variance 0.2%) from Palau. Group 2, from Fiji (variance 0%) differs by 8.7% sequence divergence from Group 1, and by over 10% from the other two haplogroups (Table 4). Group 3, from the main islands of Palau and the northernmost of the South West Islands (Sonsorol) differs by 6.7% from Group 4, which is unusual among the *Trimma milta* haplogroups in having representatives from three major geographical localities (Palau, Raja Ampat and Flores) while exhibiting only modest variance (1.3%).

**Table 4.** Results from a barcode analysis of 21 specimens of *Trimma milta*, giving maximum variation within each group followed by the minimum distances between the groups (as percentages). Pal/RA/Flores – Palau, Raja Ampat and north-eastern Flores.

Group #	Locality	n	Variation	Min. Distance			
				1	2	3	4
**1**	Moorea	4	1.1	0	8.7	7.9	9.0
**2**	Fiji	2	0	–	0	10.2	10.1
**3**	Palau	10	0.2	–	–	0	6.2
**4**	Pal/RA/Flores	5	1.3	–	–	–	0

**Comments.** Group 3 presents a complex picture. Ten specimens from Palau have a variance of 0.2%. Two samples, excluded here because their sequences exhibit several anomalies (including stop codons), differed by a minimum distance of 3.9% from the others. To date, no work has been undertaken to test the barcode results morphologically. The only observations made were on the images from the various localities. Specimens from Moorea tend to be overall dark red to orange (although a few specimens are orange-yellow), those from Raja Ampat are pinkish with less obviously outlined scale pockets, while those from Palau are predominantly yellow, with a few of them having diffuse darker bars below the eye. Clearly there is a need for a thorough examination of specimens currently assigned to this species throughout its apparent range.

### The *Trimma hayashii* group

Fig. 1H

The species was described by Hagiwara and Winterbottom (2007) from the Ryukyu Islands, Japan, with additional type specimens from Palau and Pohnpei. Our barcode analysis of 7 specimens shows two haplogroups, one from Japan, Palau and Raja Ampat (Group 1; 5; 0.4% variance) and the other represented by specimens from Palau (Group 2; 2; 0.0% variance). These two groups are separated by 4.7% sequence divergence. It is interesting that the two specimens in the second group are from Helen Reef, the isolated southern-most of the South West Islands of Palau (Fig. 2), while the three Palauan specimens in the first group are from the main Palauan islands to the north. Closer morphological analysis of the Helen Reef material assigned to this species is clearly indicated.

### The *Trimma striatum*/*Trimma capostriatum* group

Fig. 1I

These two species have long been considered synonyms (e.g. Winterbottom 1984). *Trimma striatum* was described from the Philippines by Herre (1945, as *Coronogobius*), and *Trimma capostriatum* was described by Goren (1981, as *Zonogobius*) from New Caledonia. Recent work by Hoese (as part of Winterbottom and Hoese in prep.) has enumerated colour pattern differences between the two nominal species suggesting that they are distinct species, and that conclusion is bolstered by our barcode data. Specimens of *Trimma striatum* from Palau and Raja Ampat (6; 0.9% variance) differ from specimens identified as *Trimma capostriatum* from the Great Barrier Reef and Rabaul (11; 0.3% variance) by a minimum of 9.3% sequence divergence. These two species were identified from the same collection made in the lagoon at Helen Reef, South-West Islands of Palau using colour pattern criteria, and this syntopy further supports their distinctiveness.

### The *Trimma stobbsi* group

Fig. 1J

*Trimma stobbsi* was described by Winterbottom (2001) from New Caledonia (type locality), with paratypes listed from the Maldives, Indonesia, Papua New Guinea, the Philippines and the Solomon Islands. Our barcode analysis of 11 specimens is divided into three haplogroups. The first, from Palau and Raja Ampat (Group 1; 2; 0.0% variance), differs from the other two by a minimum of 12.4%; the second group (from Palau only, Group 2; 3; 0.0% variance) differs from the third group from New Caledonia, Rabaul and Raja Ampat (Group 3; 6; 0.3% variance) by 7.9% sequence divergence. This third group probably represents the same haplogroup as the holotype. The data thus suggests that there are at least two other undescribed species in this complex. More detailed examination of the morphology and colour patterns of these specimens is needed.

### The *Trimma okinawae* group

Figs 1K, 7; Table 5

*Trimma okinawae* was described by Aoyagi (1949: 173) as a new subspecies, *Eviota caesiura okinawae*, from three specimens from Japan (the holotype, a 24 mm SL male, from Itoman, Okinawa Island). All the type specimens are apparently lost (D.F. Hoese pers. comm.).

Specimens identified morphologically as *Trimma okinawae* proved to consist of eight discreet haplogroups according to our analysis, second only to the *Trimma tevegae* group in number of haplogroups previously ascribed to a single species. A single specimen identified as this species from Palau was included in our original analysis (as Group 1, ROM T00075), but the subsample we used for sequencing appears to have been contaminated, as it was closely grouped with 10 specimens of another gobiid, *Priolepis cincta*, from several western Pacific localities. We have omitted this specimen from further consideration here, assuming that contamination of the sample has occurred.

**Figure 7.**
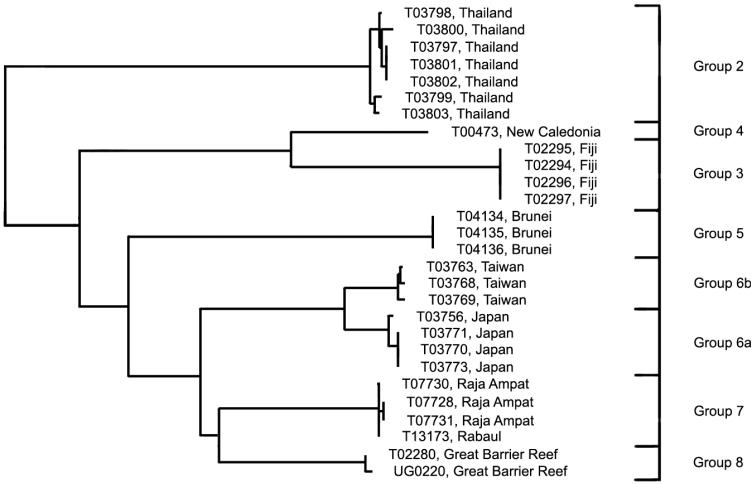
Relationships of 28 specimens of the *Trimma okinawae* group based on COI.

**Analysis.** The phenetically most distinctive haplogroup (n=7, variance 0.9%) was from Phuket, Thailand (Group 2), and this group was separated from the cluster including all the other specimens originally identified as this species in the barcode phenogram (Fig. 1). They differed from their nearest neighbour, identified as *Trimma okinawae* (Group 7, from Rabaul and Raja Ampat) by a minimum of 16.5%, and from the most divergent group (Group 3, from Fiji) by a minimum of 19.9% (Fig. 7, Table 5). Groups 3 (Fiji) and 4 (New Caledonia) are nearest neighbours and differ from each other by 7.9% and from the remaining five groups by a minimum of 14.6%. Of the latter, the specimens from Brunei (Group 5) are the most divergent, with a minimum distance of 11.6% from the remaining four groups, which are divided into two pairs, separated by a minimum of 7.8% from each other. Specimens from Japan (the type locality) differ from those from Taiwan by a minimum of 2.4%, while the specimens from Raja Ampat and Rabaul differ from those from the Great Barrier Reef by a minimum of 7.1%.

**Table 5.** Results from a barcode analysis of 28 specimens of *Trimma okinawae*, giving number of specimens, maximum variation within each group followed by the minimum distances between the groups (as percentages). Abbreviations: GBR, Great Barrier Reef; New Caledonia; Ra/RA, Rabaul and Raja Ampat.

Group#	Locality	n	Variation	Minimum distance between groups						
				3	4	5	6a	6b	7	8
**2**	Thailand	7	0.9	19.9	19.3	17.9	17.8	18.2	16.5	17.8
**3**	Fiji	4	0.0	–	7.9	17.9	16.8	16.3	17.0	16.2
**4**	New Cal.	1	n/a		–	16.4	14.8	14.6	15.6	15.3
**5**	Brunei	3	0.0			–	13.7	13.3	13.1	11.6
**6a**	Japan	4	0.3				–	2.4	8.9	7.9
**6b**	Taiwan	3	0.3					–	8.9	7.8
**7**	Ra/RA	4	0.2						–	7.1
**8**	GBR	2	0.2							–

**Comments.** Although some morphological variation in the extent of predorsal scalation, depth of interiorbital groove, length of second spine of first dorsal fin, and colour pattern—especially the vertical markings on the cheek and opercle and the overall base colour—have been noticed (D.F. Hoese pers. comm.; RW pers. obs.), no consistent differences have been recorded or published to date. However, it is planned to describe the Great Barrier Reef population as a discreet species (Winterbottom and Hoese in prep.). Again, further work, both morphological and genetic, is sorely needed to resolve the issues raised by our barcode analysis.

### The *Trimma benjamini* group

Fig. 1L

This species was described based on type specimens from the Philippines (Winterbottom 1996), and specimens identified on the basis of morphology as this species have been recorded from throughout most of the western Pacific and out onto the Pacific plate (Marshall Islands). Unfortunately, no specimens from the Philippines were available for genetic analysis. Among the 14 specimens that were available, there appear to be three haplogroups. The minimum percentage differences between these groups, while above the typical 2% sequence divergence threshold delineating different species of fishes (e.g. April et al. 2013), are relatively low compared to those found in many other haplogroups identified as a single species of *Trimma*. A group of specimens from Palau (Group 1; 4; 0.7% variance) differs from those from Raja Ampat (Group 2; 2; 0% variance) by 3.4% sequence divergence. These two groups differ from a third group from the Great Barrier Reef, New Caledonia, Palau and Rabaul (Group 3; 8; 1.3% variance) by a minimum of 5.2%. Much of the intra-group variation in the latter is encompassed between the two specimens from Rabaul, so that there appears to be little or no geographical structuring of that variation. However, further morphological work is needed to explore the apparent structure described above.

### The *Trimma emeryi* group

Fig. 1M, 8; Table 6

*Trimma emeryi* was described by Winterbottom (1985) based on numerous specimens from the Chagos Archipelago, central Indian Ocean. We have been unable to obtain specimens from the type locality for genetic analysis. The barcode analysis reveals five haplogroups identified as this species for specimens from the western Pacific, for three of which we have only a single specimen. These five groups are separated by large genetic distances.

**Figure 8.**
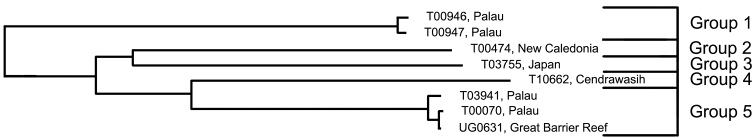
Relationships of 8 specimens of the *Trimma emeryi* group based on COI.

**Analysis.** The two specimens from Palau in Group 1 are the most divergent in COI, being separated from the other four groups by a minimum of 17.9%. Groups 2 and 3 are nearest neighbours and differ from each other by 16.1%, and from Groups 4 and 5 by a minimum of 17.2%. The latter two groups are phenetically closest to each other, differing by 13.8% (Fig. 8, Table 6). Group 5 is the only group to contain specimens from more than one locality (the Great Barrier Reef and Palau).

**Table 6.** Results from a barcode analysis of 8 specimens of *Trimma emeryi*, giving number of specimens, maximum variation within each group followed by the minimum distances between the groups (as percentages). Abbreviations: GBR, Great Barrier Reef; New Cal., New Caledonia.

Group #	Locality	n	Variation	Minimum Distance			
				2	3	4	5
**1**	Palau	2	0.5	20.1	21.9	19.7	17.9
**2**	New Cal.	1	n/a	–	16.1	17.2	18.8
**3**	Japan	1	n/a		–	18.3	18.9
**4**	Cendrawasih	1	n/a			–	13.8
**5**	GBR/Palau	3	0.8				–

**Comments.** The specimens from which genetic samples of Group 1 were taken were noted in the field as being paler in overall colouration than is usually the case for specimens identified as this species. Winterbottom (1985) had noted that specimens from Fiji differed somewhat morphologically from the Chagos type material, and suggested that further studies were necessary. Those studies have not yet been undertaken, but are obviously necessary given the barcode results presented above.

### The *Trimma caesiura* group

**Figure 9.**
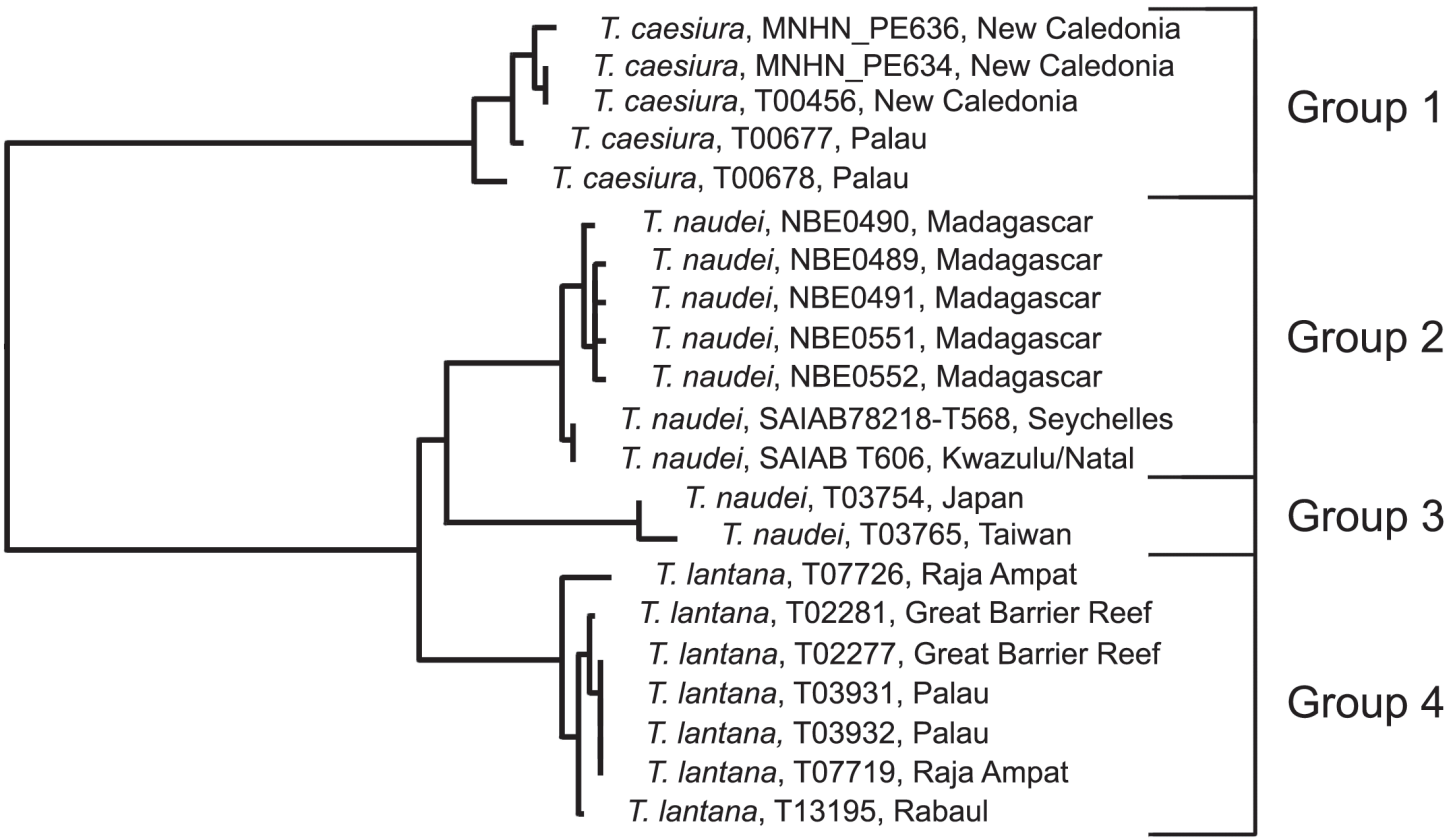
Relationships of 21 specimens of the *Trimma caesiura* group based on COI.

Figs 1N, 9, 14; Table 7

This complex was reviewed morphologically by Winterbottom and Villa (2003). In that paper, the authors included several species that are widely separated from *Trimma caesiura* (Jordan & Seale) in the barcode phenogram. We will here only consider the species currently identified as *Trimma caesiura*, *Trimma lantana* Winterbottom & Villa and *Trimma naudei* Smith.

**Analysis.** *Trimma caesiura* (5; 0.9% variance; Fig. 14A) is separated by a minimum of 10.1% from the other three groups (Fig. 9, Table 7), while *Trimma lantana* (7; 0.8% variance; Fig. 14D) is separated from *Trimma naudei* (Groups 2 and 3) by between 2.3% (Group 2, western Indian Ocean; Fig. 14B) and 4.0% (Group 3, Japan/Taiwan; Fig. 14C). The specimens identified as *Trimma naudei* from the western Indian Ocean are separated from those from Japan and Taiwan by 3.0%. Interestingly, the former of these exhibits a closer phenetic similarity to *Trimma lantana* than it does to the Japan/Taiwan specimens identified as *Trimma naudei*.

**Table 7.** Results from a barcode analysis of 21 specimens of the *Trimma caesiura* group, giving maximum variation within each group followed by the minimum distances between the groups (as percentages). Abbreviations: GBR/Pal/RA, Great Barrier Reef, Palau, Raja Ampat; NC, New Caledonia; WIO, Western Indian Ocean.

Group #	Locality	n	Variation	Min. distance		
				2	3	4
**1** (*Trimma caesiura*)	NC/Palau	5	0.9	10.5	10.7	10.1
**2** (*Trimma naudei*)	WIO	7	0.5	0	3.0	2.3
**3** (*Trimma naudei*)	Japan/Taiwan	2	0.4	–	0	4.0
**4** (*Trimma lantana*)	GBR/Pal/RA	7	0.8	–	–	0

**Comments.** Specimens identified as *Trimma naudei* range from the Comores Islands in the western Indian Ocean, through Thailand and Vietnam to Japan, and throughout Indonesia to Sulawesi and north to the Philippines. Unfortunately, we have no material for genetic analysis from these other western Pacific localities. *Trimma lantana* shows considerable morphological variation across the north of Australia from the Great Barrier Reef to as far west as Shark Bay in Western Australia. Our only genetic samples from Australia come from the Great Barrier Reef. Specimens north of the Great Barrier Reef to Raja Ampat, Rabaul, and Helen Reef (South West Islands of Palau) appear to be very uniform in colour pattern and morphology.

### The *Trimma taylori* group

**Figure 10.**
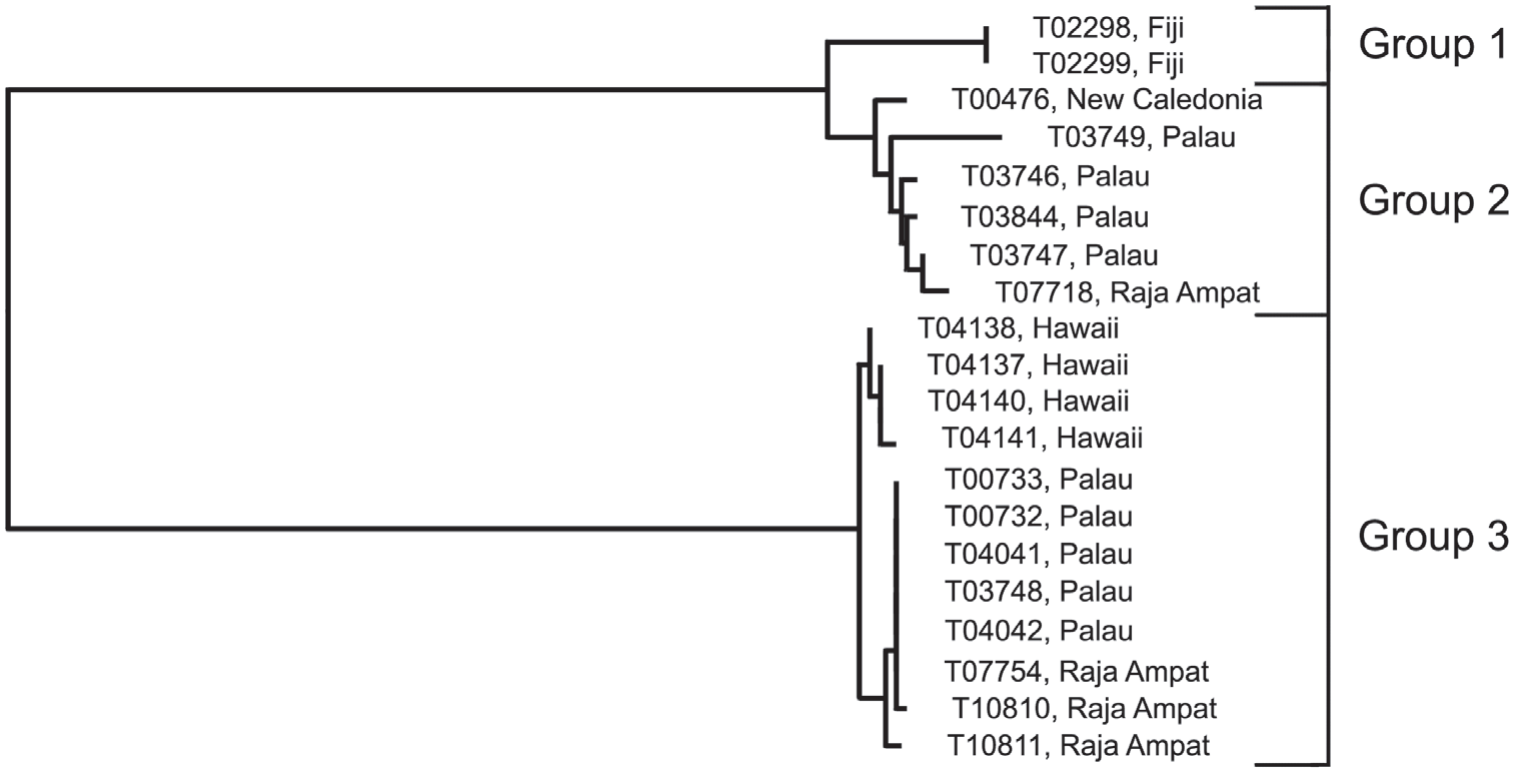
Relationships of 20 specimens of the *Trimma taylori* group based on COI.

Figs 1O, 10; Table 8

*Trimma taylori* was described from specimens collected off Oahu in the Hawaiian Islands (Lobel 1979). Specimens identified as this species based on morphology have been found from the northern Red Sea and Comores in the west through the Great Barrier Reef to the Society Islands in the east, and north to the Philippines.

**Analysis.** The barcode analysis suggests three haplogroups (Fig. 10) for specimens identified as this species on the basis of morphology and colour pattern. One of these, Group 1 (Fiji) is only marginally separated from Group 2 from New Caledonia, Palau and Raja Ampat (2.4% variance; Table 8). However, these two groups are widely separated from Group 3 from Hawaii, Palau and Raja Ampat (minimum of 17.6%).

**Table 8.** Results from a barcode analysis of 20 specimens of the *Trimma taylori* group, giving maximum variation within each group followed by the minimum distances between the groups (as percentages). Abbreviations: NC, New Caledonia; Pal, Palau; RA, Raja Ampat.

Group #	Locality	n	Variation	Min. distance	
				2	3
**1**	Fiji	2	0.0	2.4	18.8
**2**	NC/Pal/RA	6	1.7	–	17.6
**3**	Hawaii/Pal/RA	12	0.8		–

**Comments.** The four specimens from Hawaii in our analysis were collected from the same island (Oahu) that the type series is from, and Group 3 undoubtedly represents the species name. It is therefore likely that Group 2 represents an undescribed species, but there are no morphological criteria currently known that would allow specimens to be identified from museum collections. Whether the Fijian samples (here separated as Group 1) represent the same or a different species as the remaining members in Group 2 also needs to be closely examined.

### The *Trimma nasa* group

**Figure 11.**
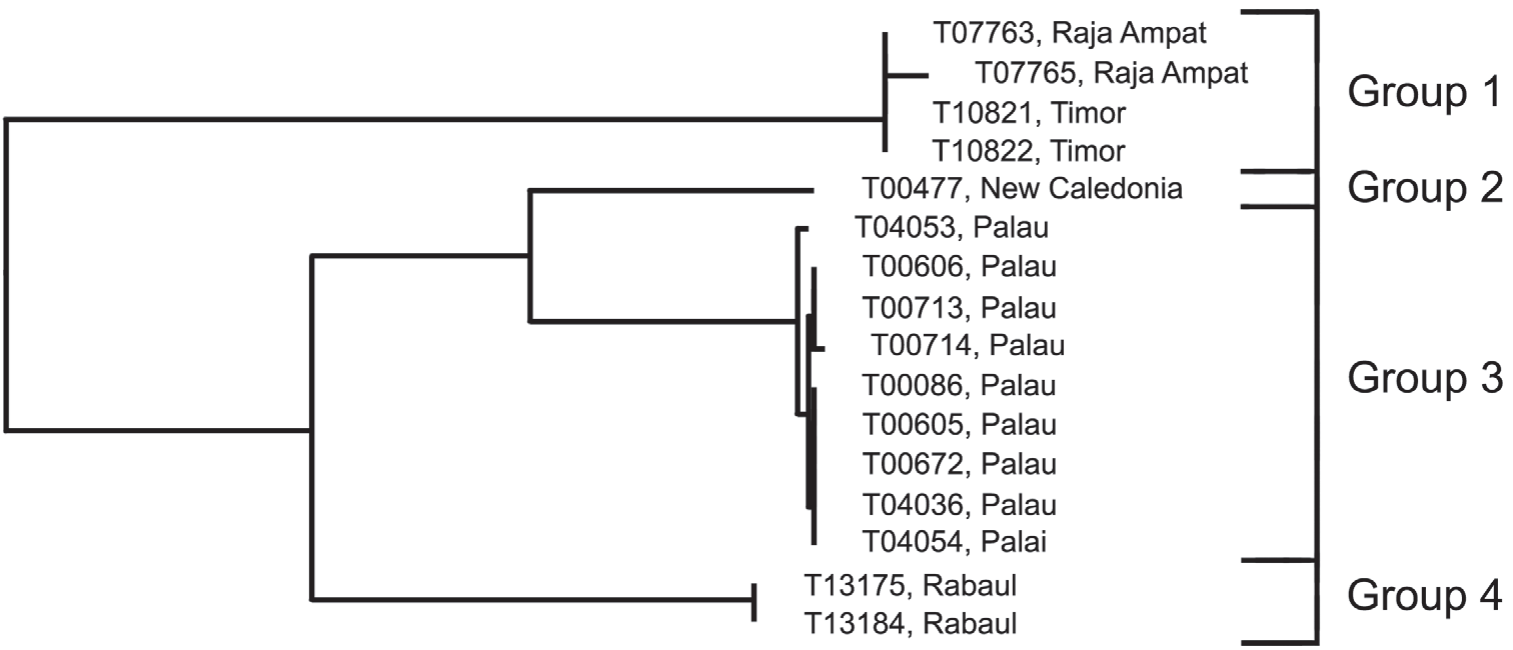
Relationships of 16 specimens of the *Trimma nasa* group based on COI.

Figs 1P, 11, 14; Table 9

*Trimma nasa* was described by Winterbottom (2005b) based on specimens from the Philippines (type locality: Siquijor Island) plus one large lot from the Solomon Islands (Guadalcanal). Additional non-type material listed was from Fiji, the Great Barrier Reef, Indonesia, New Caledonia, Papua New Guinea, Palau and Vanu Atu. Material available for this paper consisted of specimens from Raja Ampat and Timor (Indonesia: Group 1), New Caledonia (Group 2), Palau (Group 3) and Rabaul (Papua New Guinea: Group 4). Unfortunately, no specimens from the type locality were available, so it is unknown as to which, if any, of the four haplogroups recovered by our barcode analysis the types belong to. Although all our specimens are from single political entities, there may be large distances between samples. For example, specimens from Indonesia (Raja Ampat and Timor) are separated by a straight-line distance of about 1,000 kms, and those from Palau (main islands to Helen Reef) by about 570 kms (Fig. 2).

**Analysis.** Group 1 (Raja Ampat and Timor, 4; 0.4% variance; Fig. 14E) is separated from the other three groups by a minimum of 15.9% of COI (Fig. 11, Table 9). Groups 2 (New Caledonia; Fig. 14F) and 3 (Palau, 9; 0.5% variance; Fig. 14G) are nearest phenetically, and differ from each other by 5.5%, and from Group 4 (from Rabaul, 2; 0.0% variance; Fig. 14H) by a minimum distance of 9.0%.

**Table 9.** Results from a barcode analysis of 16 specimens of the *Trimma nasa* group, giving maximum variation within each group followed by the minimum distances between the groups (as percentages).

Group #	Locality	n	Variation	Min. Distance		
				2	3	4
**1**	Raja Ampat/Timor	4	0.4	16.0	16.8	15.9
**2**	New Caledonia	1	n/a	0	5.5	9.0
**3**	Palau	9	0.5	–	0	9.3
**4**	Rabaul	2	0.0	–	–	0

**Comments.** The original description of *Trimma nasa* noted that the specimens examined from Palau and New Caledonia essentially lacked the dark nasal stripe characteristic of specimens from other areas (we note in passing that specimens from these two areas have the least distance between them of any of the haplogroups uncovered in our analysis). However, further work is needed to try to uncover morphological characters that may allow for separation among the four haplogroups uncovered in our barcode analysis.

### The *Trimma marinae* group

**Figure 12.**
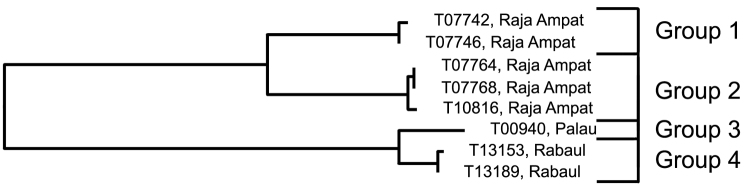
Relationships of 8 specimens of the *Trimma marinae* group based on COI.

Figs 1Q, 12; Table 10

*Trimma marinae* was described in the same paper as *Trimma nasa* (Winterbottom 2005b), based on specimens from Palau. Winterbottom (2005b) included Japan and New Britain in the distribution of the species, based on underwater photographs, and the range has since been extended to Raja Ampat (Dimara et al. 2010). Our barcode results suggest that there are four haplogroups currently identified as this species.

**Analysis.** The single sample (Group 3, Fig. 12) from the type locality, Palau, differs from those from Rabaul (Group 4; 2; 0.2% variance) by 2.0%, which is identical to the degree of difference suggesting discreet species. These two groups differ from specimens from Raja Ampat by a minimum of 15.0%. The latter are divided into two haplogroups. Group 1 (2; 0.2% variance) differs from Group 2 (3; 0.3% variance) by 4.8% sequence divergence.

**Table 10.** Results from a barcode analysis of 8 specimens of the *Trimma marinae* group, giving maximum variation within each group followed by the minimum distances between the groups (as percentages).

Group #	Locality	n	Variation	Min. Distance		
				2	3	4
**1**	Raja Ampat	2	0.2	4.8	15.0	15.0
**2**	Raja Ampat	3	0.3	0	15.6	15.3
**3**	Palau	1	n/a	–	0	2.0
**4**	Rabaul	2	0.2	–	–	0

**Comments.** There are currently no known morphological or colour pattern differences supporting these different haplogroups.

**Figure 13.**
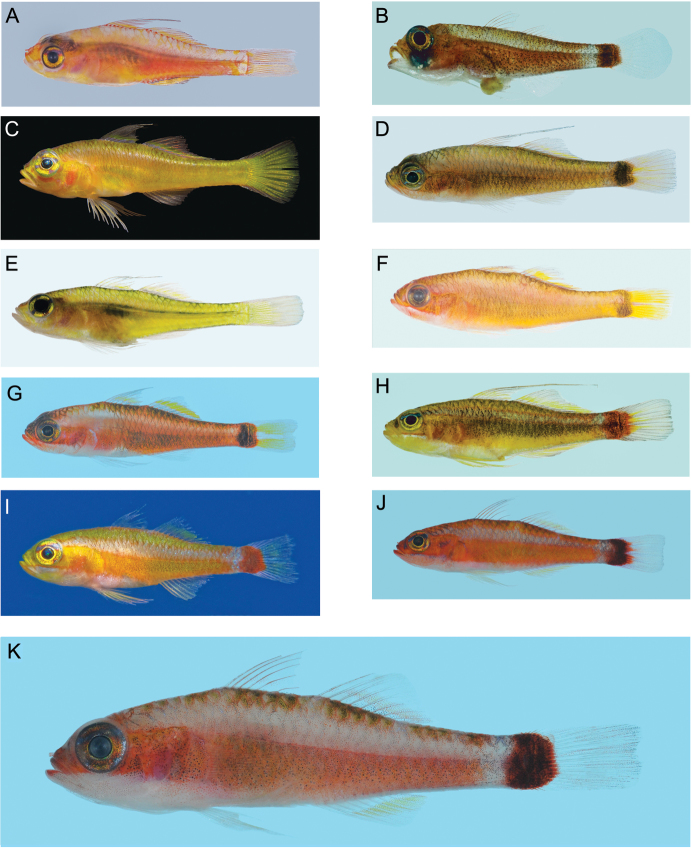
Left lateral views of freshly collected specimens of 10 of the 11 haplogroups forming the *Trimma tevegae* group (no image available for *Trimma xanthochrum* Group 2a, from Ceram), plus the phenetically basal taxon, *Trimma habrum*. All images by R. Winterbottom, except for C and I (courtesy of Mark V. Erdmann). Standard length, sex, locality and catalogue number given where available. **A** *Trimma habrum*, 16.8 male, Penemu I., Raja Ampat, ROM 84881 **B** *Trimma tevegae* (Group 1), 14.4 female, Uchelbeluu Reef, Palau, ROM 80390 (note prolapsed intestine) **C** *Trimma gigantum* (Group 9), Fam Is., Raja Ampat **D** *Trimma xanthochrum* (Group 2c), 19.8 male, Uchelbeluu Reef, Palau, ROM 93075 **E** *Trimma gigantum* (Group 8), 27.7 female, Uchelbelu Reef, Palau, ROM 80353 **F** *Trimma xanthochrum* (Group 2), 22.0 female, Penemu I., Raja Ampat, ROM 84885 **G** *Trimma xanthochrum* (Group 2b), 18.4 female, Rabaul, New Britain, ROM 88170 **H** *Trimma tevegae* (Group 5), 22.5 male, Koror, Palau, ROM 80312 **I** *Trimma tevegae* (Group 4), SE Misool, Raja Ampat **J** *Trimma caudomaculatum* (Group 7), 21.5 female, Rabaul, ROM 92109 (Note broken tip of second spine of first dorsal fin) **K** *Trimma tevegae* (Group 6), 16.0 male, Rabaul, ROM 92319 (group 6 is believed to represent the ‘true’ *Trimma tevegae*).

**Figure 14.**
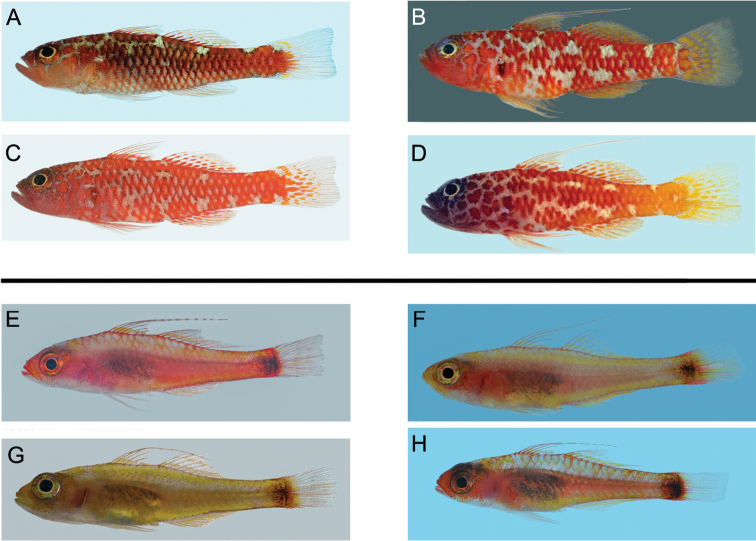
Left lateral views of freshly collected specimens of the four haplogroups forming the *Trimma caesiura* group (above), and the four haplogroups of the *Trimma nasa* group (below). All images by R. Winterbottom. Standard length, sex, locality and catalogue number follow the species name. **A** *Trimma caesiura*, 24.0 male, Babeldaob I., Palau, ROM 76105 **B** *Trimma naudei*, 26.7 male, Comores, ROM 59796 **C** *Trimma naudei*, 23.3 female, Nha Trang, Vietnam, ROM 73199 **D** *Trimma lantana*, 22.6 male, Helen Reef, Palau, ROM 83077 **E** *Trimma nasa*, 19.5 female, Kepotsol I., Raja Ampat, ROM 85321 **F** *Trimma nasa*, 22.6 female, New Caledonia, ROM 63925 **G** *Trimma nasa*, 16.5 female, Uchelbeluu Reef, Palau, ROM 80392 **H** *Trimma nasa*, 16.7 female, Rabaul, New Britain, ROM 92157.

## Discussion

The results we have presented suggest that there are nearly double the number of ‘species’ originally recognized in our analysis (52 versus 94) if one accepts the guideline that a 2% barcode sequence divergence between two groups roughly equates to different species (April et al. 2013). Our results differ somewhat from previous barcode studies in other genera of gobies. For example, although our findings are comparable in the magnitude of the differences we found among *Trimma* lineages (despite the fact that we used minimum distances between lineages rather than the mean distances reported by both Tornabene et al. 2010 and Baldwin et al. 2009), we often uncovered a greater percentage of cryptic species within the groups analysed above (e.g. for *Trimma okinawae*). This is not surprising, given the far larger area encompassed by the Indo-Pacific (vs. the Caribbean), and the more intensive traditional systematic studies of the latter area. We note that our results based on recognizing groups based on a 2% or greater difference in COI differ from a RESL/BIN analysis we performed on an updated data set in only one case. This was the recognition by the latter method of two groups of what we consider here to be *Trimma caudomaculatum* – one from Rabaul, and the other from Japan. Interestingly, 5 of the paratypes of this species from Japan have a few branched rays in the pectoral fin (2–5 branched, mean 3.0, n=5) whereas these rays are all unbranched in the Rabaul specimens (n=4).

Our study included samples identified as belonging to 48 named and four unnamed species. The barcode results suggest that there are 94 haplogroups separated by 2% or more sequence divergence. If future research bears out the barcode results, and assuming further that RW’s estimate of the undescribed species is relatively accurate, the final number of species in the genus would be of the order of 200. Given that intensive sampling for both morphological and genetic samples of *Trimma* is lacking for the majority of Indo-Pacific coral reefs, even this number (200) may be a significant underestimate. We were unable to obtain specimens of 25 of the recognized described species (34%), and therefore we do not feel it appropriate to present the statistics found in other studies for comparative purposes (e.g. average distance between closest taxa). We are also loathe to further dissect the distance data between members of the groups we recognize above, both because these groups are not necessarily monophyletic (our analysis is based on a Neighbour-Joining network, which cannot provide phylogenetic inferences), and because we are convinced that many, if not most, of these groups contain as yet unsampled lineages which could reduce the distance values we obtained. The lack of comprehensive sampling over the vast area of the tropical Indo-Pacific, while understandable, is nevertheless frustrating.

The problem of cryptic species has been a long-standing one for biologists. With the advent of barcoding, the BOLD platform and BINs, hints of their existence have become more easily uncovered and thus available for further exploration. While the results presented in this paper may be preliminary, there are simply not enough taxonomists to check the large number of questions raised by our analysis against morphological attributes. However, our study has revealed a plethora of often very different haplogroups currently masquerading under a single specific name. The differences between these genetic lineages frequently exceeds (sometimes dramatically so) the guide-lines of a 2% difference in the barcode sequence as being equivalent to a species. Our purpose, then, is to draw attention to the magnitude of the problem facing any inventory of gobiid species (especially of *Trimma*, and potentially other small and very speciose genera such as *Eviota*), and to expose the exciting potential that further and more wide-ranging research into certain recalcitrant groups within these genera could provide in terms of biogeograpy, evolution and conservation biology.
